# Overlapping Hyperpigmented and Poikilodermatous Mycosis Fungoides

**DOI:** 10.1155/crdm/6659374

**Published:** 2026-06-26

**Authors:** Joan Somja, Arjen F. Nikkels

**Affiliations:** ^1^ Department of Dermatopathology, CHU of Sart Tilman, University of Liège, Liège, B-4000, Belgium, ulg.ac.be; ^2^ Department of Dermatology, CHU of Sart Tilman, University of Liège, Liège, B-4000, Belgium, ulg.ac.be

**Keywords:** cutaneous T-cell lymphoma, hyperpigmentation, mycosis fungoides, poikiloderma

## Abstract

Primary cutaneous T‐cell lymphomas (pCTCLs) are a heterogeneous group of rare clonal T‐cell disorders, among which mycosis fungoides (MF) is the most common subtype. Several clinicopathologic variants of MF have been described, including poikilodermatous and hyperpigmented forms, with occasional overlap between variants. We report a 68‐year old man with a 25‐year history of slowly progressive, asymptomatic dark plaques involving the lower extremities and intergluteal fold consistent with overlapping hyperpigmented and poikilodermatous MF. Unlike the classically reticulated appearance of poikilodermatous MF, the lesions displayed extensive, sharply demarcated confluent hyperpigmentation. 18F‐FDG PET/CT demonstrated no systemic involvement, although the cutaneous lesions displayed mild hypermetabolism. Histopathological examination revealed epidermotropic atypical lymphocytes with superficial dermal lymphohistiocytic infiltrates, vascular ectasia, erythrocyte extravasation, hemosiderin deposition, and dermal melanophages. Immunophenotypic studies supported the diagnosis of MF with monoclonal T‐cell receptor rearrangement. Disease staging was T2N0M0B0 (Stage IB). Given the longstanding indolent course, absence of symptoms, and lack of extracutaneous disease, a watch‐and‐wait strategy was adopted. Although hyperpigmentation may be observed in poikilodermatous MF, the unusually marked and confluent pigmentation in our patient exceeded the degree typically associated with poikiloderma alone, suggesting overlap with the hyperpigmented variant of MF. This case highlights the importance of careful clinicopathologic correlation in atypical pigmentary presentations of MF.

## 1. Introduction

Primary cutaneous T‐cell lymphomas (pCTCL) comprise a heterogenous group of rare T‐cell clonal disorders with a large range of clinical presentations [1, 2]. Mycosis fungoides (MF) is the most frequently observed subtype, representing around 70% of the cases [1–3].

In general, most patients with MF will have an indolent disease course, although cases of MF‐related death are not uncommon, even in early‐stage disease. The most frequent cause of death in these patients is pulmonary infection, linked to the Th2 immune profile, followed by cardiovascular failure and blastic transformation [4].

MF is classified into the classic form, which is the most frequently encountered (60%–70% of the cases), and a high number of several less common variants such as follicular MF, syringotropic MF, granulomatous slack skin, and pagetoid reticulosis [1, 5]. Even rarer presentations include poikilodermatous MF (pMF) [6] and the hypo [7, 8] or hyperpigmented MF (hMF) subtypes [9–12]. In uncommon cases, overlap between different variants may occur, although this does not typically seem to influence therapeutic decisions or treatment response.

A unique case of MF is presented in a man with several unusual features, including a very longstanding history of concurrent poikilodermatous and markedly hyperpigmented MF (phMF), with pigmentation far more prominent than typically observed in classical pMF. The unusually intense hyperpigmentation partially obscured the characteristic reticulated poikilodermatous features, creating an atypical clinicopathologic presentation suggestive of overlap between both variants and representing a potential diagnostic pitfall for clinicians unfamiliar with these uncommon MF subtypes. Despite extensive body surface involvement, the patient remained free of pruritus, systemic symptoms, and extracutaneous involvement throughout the disease course, further illustrating the indolent nature that may characterize these uncommon MF variants.

## 2. Case Presentation

A 68‐year old man with no other significant medical, surgical, or allergic history presented of at least a history of 25 years of very slowly progressive markedly hyperpigmented cutaneous plaques, nonsclerotic, noninfiltrated, and unaccompanied by erythema or pruritus. Minimal scaling of the plaques was noted. His daily medication included aspirin 80 mg/day and simvastatin 40 mg/day. Previous topical treatments prescribed in primary care, including emollients and high potency topical corticosteroids, had produced no meaningful improvement. Due to the absence of pruritus and other cutaneous or systemic signs, the patient had not sought earlier specialist evaluation. Three years earlier, a dermatologist had performed a punch biopsy suggestive of MF, characterized by some atypical epidermotropic lymphocytes, an immunohistochemical profile including CD3^+^, CD4^+^, CD45RO^+^ epidermotropic T‐cell infiltrate with loss of CD7 and pigment incontinence, and a monoclonal T‐cell receptor (TCR) rearrangement. However, no further workup or staging was undertaken. The patient wished a second opinion and was therefore recently referred to a skin cancer clinic for diagnostic and staging workup as well as therapeutic management.

**FIGURE 1:**
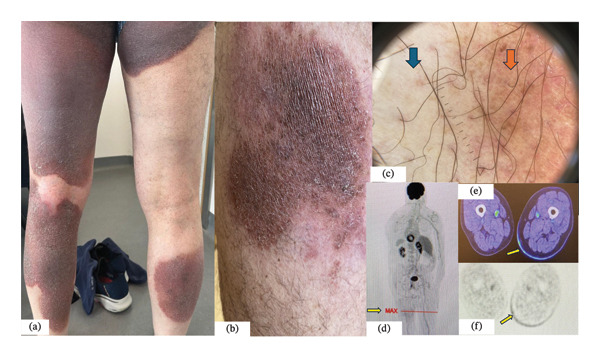
Clinical presentation and 18F‐FDG PET/CT imaging features. (a) Well‐circumscribed, reticulated, coalescent, nonpruritic dark red‐to‐brown patches involving the lower extremities. (b) Close‐up view of the poikilodermatous and hyperpigmented lesion of the anterior aspect of the right thigh. (c) Dermoscopic image revealing the typical features of poikiloderma blue arrow: normal skin, red arrow: poikilodermatous skin. (d) ^18^F‐FDG PET/CT imaging demonstrating focal hypermetabolic uptake within the thigh and lower extremity lesions (yellow arrow). Maximum intensity projection image showing hypermetabolic activity in the thigh. (e) Axial fused PET/CT image demonstrating focal subcutaneous hypermetabolic uptake in the thigh (yellow arrow). (f) Corresponding axial PET image confirming focal hypermetabolic activity (yellow arrow).

Clinical examination revealed extensive, well‐demarcated, coalescent dark red‐to‐brown hyperpigmented plaques involving the lower extremities, with focal reticulated and poikilodermatous areas (Figure 1a, b). Lesions also involved the intergluteal fold, with no other body sites affected. Dermoscopy of the border of the lesions revealed a reticular network of telangiectasia, atrophy, and pigmentary alteration, with a mottled red‐brown appearance (Figure 1c). No lymphadenopathy was detected, and the remainder of the physical examination was unremarkable. Diagnostic workup included ^18^F‐FDG PET/CT, repeat cutaneous biopsy, and blood studies. PET/CT demonstrated no suspicious extracutaneous lesions or lymphadenopathy, although the cutaneous lesions of the thighs showed mild hypermetabolism (Figure 1d–f).

**FIGURE 2:**
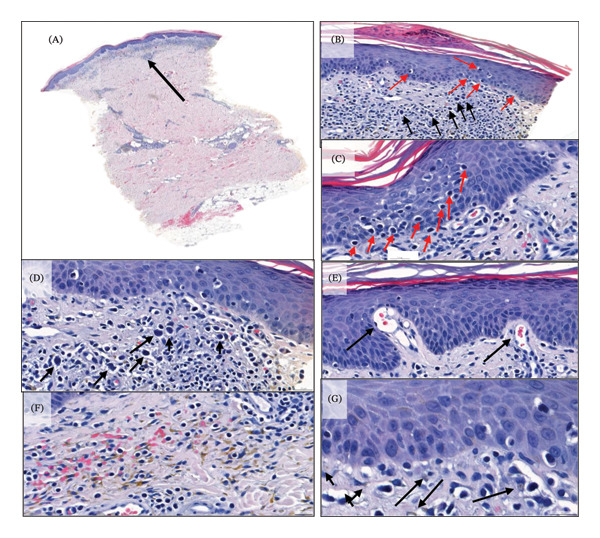
Histopathological and immunohistochemical features of poikilodermatous mycosis fungoides. (A) Superficial dermal lymphocytic infiltrate (H&E, × 1.25). (B) Epidermotropism without spongiosis (red arrows) and lymphocytic atypia (black arrows) (H&E, × 20). (C) Higher‐magnification epidermotropism (red arrow) (H&E, × 40). (D) Atypical lymphocytes (black arrow) (H&E, × 40). Poikilodermatous changes including superficial dermal vascular ectasia ((E), black arrow), erythrocyte extravasation with hemosiderin deposition (F), and subtle pigmentary incontinence with dermal melanophages ((G), black arrows) (H&E, × 20–80).

The hematoxylin‐eosin (H&E) histopathological examination of the cutaneous biopsy demonstrated epidermotropic atypical lymphocytes without associated spongiosis together with a superficial band‐like dermal lymphohistiocytic infiltrate (Figure 2A–D). The atypical lymphocytes were enlarged, irregularly contoured, and occasionally displayed subtle cerebriform nuclei. Additional findings included superficial dermal vascular ectasia, erythrocyte extravasation with prominent hemosiderin deposition, and focal pigmentary incontinence with numerous dermal melanophages, supporting poikilodermatous changes (Figure 2E–G). Classical histological features of poikiloderma, including epidermal atrophy, dermal fibrosis, and solar elastosis, were relatively inconspicuous, likely reflecting biopsy sampling from a clinically thicker plaque area rather than a more atrophic portion of the lesion.

**FIGURE 3:**
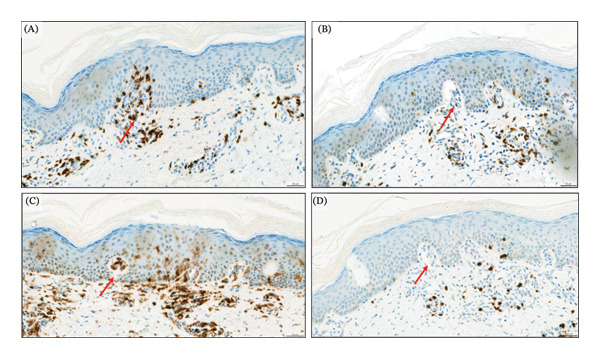
Immunohistochemical studies (DAB, × 20) showing epidermotropic atypical T lymphocytes positive for CD3 (A) and CD4 (B), with partial CD7 downregulation (C) and absence of significant CD8 expression, except for occasional reactive T‐cells (D). Red arrows indicate the same epidermotropic focus for comparison across immunostainings.

Immunohistochemical studies showed an epidermotropic T‐cell infiltrate positive for CD3 and predominantly expressing CD4, without significant loss of pan–T‐cell markers, including CD2, CD3, CD5, and CD7, although focal attenuation of CD7 expression was noted. CD8 highlighted only occasional admixed reactive T lymphocytes (Figure 3A–D), and focal CD30 expression was observed. Overall, the clinicopathologic and immunophenotypic findings supported the diagnosis of an hMF and pMF. No TCR rearrangement was evidenced.

No circulating Sézary cells were observed, lymphocyte immunophenotyping was unremarkable, and bone marrow examination showed no abnormalities. Correlation of clinical and pathological findings established the diagnosis of concurrent phMF, staged as T2N0M0B0 (Stage IB). Given the long‐standing indolent course, absence of symptoms, and lack of systemic involvement, the multidisciplinary tumor board (dermato‐oncologist, dermatopathologist, hematologist, and radiotherapist) elected a watch‐and‐wait strategy, which was acceptable to the patient. An annual follow‐up was initiated.

## 3. Discussion

Poikilodermatous and hyperpigmented variants of MF may represent a diagnostic challenge because of their indolent course and broad clinical spectrum. Extensive hyperpigmented plaques may obscure the characteristic poikilodermatous features and delay recognition of the underlying lymphoproliferative disorder. The differential diagnosis of longstanding diffuse hyperpigmented indolent plaques includes metabolic disorders such as hemochromatosis or ochronosis; drug‐induced hyperpigmentation related to antimalarials, amiodarone, minocycline, or chemotherapeutic agents; and dermatologic conditions including scleroderma, postinflammatory hyperpigmentation, lichen planus pigmentosus, ashy dermatosis, and chronic venous stasis.

Among pCTCL, MF is the most common type and presents a large array of clinical variants [1, 2]. pMF is a patch‐stage form of MF with a large array of cutaneous alterations such as epidermal atrophy, mottled‐reticular coalescing erythematous papules and/or plaques, light‐to‐dark brown, often mottled‐reticular, hyperpigmentation, and telangiectasia [2, 6, 13]. pMF typically involves the major flexural areas and the trunk. Three clinicopathologic patterns of pMF are commonly recognized in the literature: generalized pMF, localized pMF, and pMF associated with other MF variants [6].

Our patient demonstrated overlapping clinical and histopathological features of pMF and hMF. Although hyperpigmentation may occur in pMF, the unusually extensive, confluent, and sharply demarcated pigmentation observed in our case exceeded the degree typically expected in poikiloderma alone, while the characteristic reticulated appearance remained relatively subtle. Histopathological examination nevertheless demonstrated several features supporting poikilodermatous change, including vascular ectasia, erythrocyte extravasation, hemosiderin deposition, and dermal melanophages.

Several previously reported cases of hMF have similarly highlighted the diagnostic difficulty posed by longstanding pigmentary dermatoses. Böer‐Auer et al. described a patient initially treated for lichen planus pigmentosus for several years before repeated biopsies, and clinicopathologic correlation established the diagnosis of hMF, emphasizing the overlap between interface dermatitis, pigment incontinence, and epidermotropic atypical lymphocytes [14]. Likewise, Zha and Lu recently reported a 17‐year diagnostic evolution from lichen planus pigmentosus to hMF, underlining the importance of repeated histopathological assessment in persistent or progressive hyperpigmented eruptions [11]. In the retrospective series by Jung et al., hMF represented approximately 10% of MF cases and frequently coexisted with other atypical variants, including poikilodermatous lesions [9]. Like our patient, most reported cases followed an indolent clinical course and presented without extracutaneous involvement [9, 11, 13]. However, the sharply demarcated and extensive confluent hyperpigmentation observed in our case appeared particularly prominent relative to the usually more reticulated pigmentation described in pMF [6, 14].

Unlike several reported cases of hMF associated with atypical immunophenotypes, particularly CD8‐predominant or CD4/CD8 double‐negative profiles [13], our patient showed a predominantly CD4‐positive epidermotropic T‐cell infiltrate. Interestingly, hMF has been reported to present predominantly at early stages, with approximately 85.7% of patients diagnosed with Stage IA–IIA disease [13]. Overall, hMF may coexist with other MF variants, and our case further illustrates how marked hyperpigmentation may dominate the clinical presentation and partially obscure the expected reticulated poikilodermatous features.

Both pMF and hMF appear to follow a more indolent course and carry a more favorable prognosis than classic MF [14, 15]. In our case, the patient had a longstanding course exceeding 25 years, without manifestations other than the cutaneous lesions, notably longer than the 17‐year disease duration reported by Zha and Lu [11]. Therefore, aggressive treatment strategies should generally be avoided. No specific treatment guidelines are available for pMF and hMF; management should follow recommendations for early‐stage MF [15, 16]. As no systemic involvement was identified in our patient, skin‐directed therapies represented the preferred first‐line approach. Ultraviolet (UV)‐based therapies, particularly narrowband UVB phototherapy, are commonly recommended for both pMF and hMF, although marked hyperpigmentation may reduce the efficacy of light‐based treatments. Other therapeutic options, including topical corticosteroids, localized radiotherapy, and 308‐nm excimer laser therapy, may also be considered [15, 16]. While these modalities may have an action on disease activity, they are often less effective in reducing the associated hyperpigmentation. Our patient declined treatment because of the absence of pruritus, lack of disease progression, and limited concern regarding the cosmetic appearance of the lesions.

In summary, pMF and hMF may coexist and generally follow an indolent clinical course. This case is of particular interest because the pigmentary component dominated the clinical presentation, making recognition of the underlying poikilodermatous pattern more challenging. Clinicians should, therefore, be aware of these atypical presentations of MF [17], and careful clinicopathologic correlation remains essential for accurate diagnosis. When treatment is required, skin‐directed therapies remain the first‐choice approach.

## Funding

No funding has been obtained for this case report.

## Consent

Written permission was obtained from the patient for the publication of the clinical image.

## Conflicts of Interest

The authors declare no conflicts of interest.

## Data Availability

The data that support the findings of this study are available on request from the corresponding author. The data are not publicly available due to privacy or ethical restrictions.
